# Cross-Cultural Adaptation, Reliability, and Validity of a Brazilian of Short Version of the Posttraumatic Diagnostic Scale

**DOI:** 10.3389/fpsyg.2021.614554

**Published:** 2021-04-23

**Authors:** Cláudia de Faria Cardoso, Natalia Tiemi Ohe, Vera Lúcia Taba, Tamyres Tomaz Paiva, Ovidiu Constantin Baltatu, Luciana Aparecida Campos

**Affiliations:** ^1^Institute of Biomedical Engineering at Anhembi Morumbi University, Sao Jose dos Campos, Brazil; ^2^Center of Innovation, Technology and Education (CITE) at Sao Jose dos Campos Technology Park, Sao Jose dos Campos, Brazil; ^3^Hospital São Francisco de Assis, Jacareí, Brazil; ^4^Federal University of Paraiba, Joao Pessoa, Brazil; ^5^College of Medicine and Health Sciences, Khalifa University, Abu Dhabi, United Arab Emirates; ^6^College of Health Sciences, Abu Dhabi University, Abu Dhabi, United Arab Emirates

**Keywords:** posttraumatic diagnostic scale, cross-cultural adaptation, Brazil, content validity coefficient, receiver operating characteristic (ROC) curve analysis

## Abstract

**Background:** A short version of the Posttraumatic Diagnostic Scale (PDS) comprising only re-experiencing symptom items has been recently validated on Japanese adults. This short-version-PDS had good psychometric properties among Japanese adults with and without posttraumatic stress disorder (PTSD). The aim of this study was to translate and culturally validate the short-version-PDS for the Brazilian sociolinguistic context.

**Methods:** A translation of the short-version-PDS was performed based on established guidelines. We enrolled 53 patients with PTSD as a potential comorbidity. The translation and cross-cultural adaptation of the short-version-PDS included forward and back-translation by a Japanese Brazilian researcher and a certified translator; synthesis was achieved by consensus, backward translation, pilot test, and finalization. Content validity coefficient (CVC) was used to assess quality of adaptation. Internal consistency was calculated using Cronbach's alpha coefficient. Spearman correlations were between the new short-version-PDS and the Brazilian version of the posttraumatic Stress Disorder Checklist for DSM-5 (PCL-5), and a receiver operating characteristic (ROC) curve was used to determine the best cut-off values for the short-version-PDS.

**Results:** The short-version-PDS was well accepted by all subjects, none of the questions were experienced as inappropriate, and all questions of the 3 items were judged important. Item 1 presented CVCt = 0.92; item 2 had a CVCt = 0.87 and item 3 had a CVCt = 0.95. The internal consistency of the final version as measured by Cronbach's alpha was 0.78. The short-version-PDS scale correlated positively with the DSM-5 scale with a Spearman rho of 0.64 (95%CI [0.4-0.8], *p* < 0.001). The receiver operating characteristic (ROC) curve value was 0.97 (95%CI [0.9-1.0], *p* < 0.001). The cut-off score for a maximum Youden Index of 0.8 to distinguish moderate from severe from slight PTSD was > 31.0 with sensitivity and specificity are 86.4 and 93.5%, respectively.

**Conclusions:** This Brazilian Portuguese version of the short-version-PDS had good psychometric properties among Brazilian adults with and without PTSD. Transferability and generalizability of the cut-off scores should be further analyzed.

## Background

Mental health has been the focus of increasing attention due to the risks of those exposed to emergency settings, as they may be affected by physical and mental disorders, such as burnout and posttraumatic stress disorder (PTSD) (Carmassi et al., 2020). Mental health has been identified as a research area with potential benefits for research prioritization and policy setting in both the COVID-19 pandemic and future large-scale public health crises (Liu et al., 2020). The COVID-19 pandemics caused striking prevalence rates of symptoms of depression and anxiety (Lai et al., 2020; Perlis, 2020; Tan et al., 2020).

Post-traumatic stress disorder (PTSD) has consequences that can lead to poor quality of life and increased use of health and social services (Atwoli et al., 2015; Bothe et al., 2020). It is a problem that can affect people who directly experience or witness potentially stressful situations (Association Psychiatric Association, 2013), and it is twice as common in women than in men (Yehuda et al., 2015). The overall lifetime prevalence of PTSD reported in the World Mental Health Surveys studies was 3.9% for a randomly selected trauma (Kessler et al., 2017). The prevalence of PTSD in the Brazilian population, as well as has been reported over the years and in other countries, varies according to the social, economic, and demographic context (Ribeiro et al., 2013). Despite being a highly prevalent disorder, it is commonly underdiagnosed (da Silva et al., 2019). Early diagnosis can favor treatment, reducing the impairment in the daily activities of the affected person. Screening tools can be facilitators in the search for diagnosis (Price et al., 2016). Screening scales are especially useful when time is short and demand is high, as is the case in situations of natural disasters, accidents, war conflicts and pandemics. In cases like these, time and resources are scarce and short scales are even more advantageous (Itoh et al., 2017b). While self-report instruments cannot be used for diagnosis, the availability of brief assessment tools often determines whether or not a condition is assessed. For example, a two-item depression screening instrument (Löwe et al., 2005) has significantly improved the frequency with which the disorder is evaluated. Selecting the most appropriate validated collection tool for measurable outcomes to use is essential for successful patient-centered healthcare (Botero et al., 2016). For this, limiting the number of data collection points through developing and validating short versions of surveys/questionnaires to prevent survey fatigue are of interest. Short PTSD scales are being developed screening tools that are best suited to the primary care setting and for those re-experiencing PTSD symptoms (Spoont et al., 2013).

A short version of the Posttraumatic Diagnostic Scale (PDS) comprising only re-experiencing symptom items has been recently validated on Japanese adults (Itoh et al., 2017a). This Japanese short version of the Post-Traumatic Diagnosis scale (short-version-PDS) was developed based on the symptoms of trauma re-experience, because it considers them as differential for the diagnosis, because it is more related to the general severity of the symptoms and because other symptoms are ambiguous for the diagnosis (Itoh et al., 2017b). The scale consists of three items with a Likert scale for the intensity of the symptom mentioned in each item. The Japanese short-version-PDS 3-item scale has good psychometric properties among Japanese adults with and without PTSD (Itoh et al., 2017a) and has been utilized to build prediction models for depressive symptoms in large population studies (Takahashi et al., 2020). This short-version-PDS 3-item scale comprising “intrusive images (B1),” “nightmares (B2),” and “physiological reactions when reminded of the trauma (B5),” and the 2-item scale of “nightmares (B2)” and “physiological reactions when reminded of the trauma (B5)” had the highest AUCs and were generally higher than were those for previous short scales to diagnose the PTSD severity score (Itoh et al., 2017a).

Since the Japanese short-version-PDS is highly correlated with a PTSD severity score and has good psychometric properties among adults with and without PTSD (Itoh et al., 2017b), the aim of this study was to translate and culturally validate the Japanese short-version-PDS for the Brazilian sociolinguistic context.

## Methods

### Ethical Approval

Permission to translate the scale was obtained from the original author, Itoh et al. (2017a). All procedures performed in studies involving human participants were in accordance with the ethical standards of the institutional and/or national research committee and with the 1964 Helsinki declaration and its later amendments or comparable ethical standards. The study was approved by the Medical Ethics Committee of Anhembi Morumbi University in accordance with resolution 466/2012 and 340/2004 of the National Health Council (Ministry of Health) for research on human beings (CAAE 13494719.7.0000.5492).

### Study Participants

All subjects participating to the study were recruited from voluntary patients at the Hospital São Francisco de Assis, Jacareí—SP, Brazil. The final version of the Portuguese translation of the short-form PDS was extended to 53 volunteers who were referred to the clinical psychology unit from other clinical units on the basis of potentially traumatic experience. Only volunteers with at least one potentially traumatic experience were included. Patients with PTSD as a potential comorbidity, such as pregnancy loss, cancer diagnosis, and end-stage renal disease patients on hemodialysis, were recruited from the Hospital São Francisco de Assis' maternity, oncology, and hemodialysis clinics. The research was performed from September 2019 to June 2020. Participants were aged 19-66 years (Mean = 32.37; SD = 10.06), most were female (88.4%), married or cohabiting (69.8%), with completed high school (69.8%). Retrospective baseline demographic information and clinical data were collected. Patient-level data were anonymized by removing all patient-identifying details and allocating a unique study code to each recording. Written informed consent was obtained from all subjects.

### Instruments: Short-Version-PDS and DSM-5 Scales

The short-version-PDS is a self-assessment tool that seeks to investigate the intrusive symptoms that define the diagnosis of PTSD in adults (Itoh et al., 2017b). It consists of a list of potentially traumatic events and contains three of the five statements listed in DSM-5 “Diagnostic Criterion B.” The instruction is to consider the symptoms perceived in the last thirty days. To assess the severity of the symptom, short-version-PDS involves a Likert-type scale, with four response options, ranging from 0 to 3, corresponding to “No time / Only once,” “Less than once a week/Sometimes,” “Two to four times a week/Many times,” “Five or more times a week/Almost always.” The scale has a minimum score of 0 points and a maximum of 9 points.

To test the short-version-PDS translation in Brazilian, the Brazilian version of the posttraumatic Stress Disorder Checklist for DSM-5 (PCL-5) was used as a comparison (Osório et al., 2017). The PCL-5 consists of 20 items that are used to test for PTSD symptoms as described by the DSM-5. Respondents use a 5-point scale (0–4) to show how much the symptom bothered them in the previous month for each of the 20 things, ranging from “not at all” to “extremely.” A cutoff point of 36 presented the higher overall efficiency for predicting a PTSD diagnosis (Pereira-Lima et al., 2019).

### Translation and Cultural Adaptation

The method adopted for the cross-cultural adaptation of the screening scale was based on the model proposed by Beaton et al. (2000). Three translations from Japanese into Portuguese Brazilian were carried out by bilingual translators. The first translator was instructed on the research. The other translators were unaware of the research and carried out the translation in order to find cultural, semantic, and idiomatic adaptations. The consensus of the translations was reached, and the result was analyzed by two of the translators, one with knowledge of the research, the other not. Then, back-translation and pilot test were performed with six volunteers. The final analysis was carried out by a committee of health experts with experience in assisting people with a traumatic history. The panel of experts were professionals with training in the medical, nursing, occupational therapy, psychology and social programs.

For theoretical analysis of the 3 items of the instrument, the experts evaluated the 3 translations and the synthesis under the criteria of the content validity technique. The evaluation criteria were semantic, linguistic, cultural, conceptual, clarity, and precision (Beaton et al., 2000). The content validity coefficient (CVC) was calculated for each item of the instrument and for the instrument as a whole (CVCt) (Filgueiras and Hall, 2017). The CVC values accepted to consider the quality of an aspect or item judged must be >0.80 (Filgueiras and Hall, 2017).

The panel of experts measured the translations on a Likert scale ranging from 1 “very poor” to 4 “very good” in each of the items of short-version-PDS in order to assess the level of adequacy of the translation in relation to the proposed listed points. The questionnaires were applied in a private hospital, with mixed clientele (SUS, private, plans).

### Data Analysis

Internal consistency refers to how accurately survey or test items intended to evaluate the same construct actually do so. Internal consistency was assess using Cronbach's alpha coefficient which is a measure of scale reliability (MEDCALC, MedCalc Software Ltd., Ostend, Belgium, www.medcalc.org). An acceptable internal consistency, defined by Cronbach's alpha coefficient, ranged from 0.7 to 0.95 (Bland and Altman, 1997).

The validity of a test depends on the hypothesis presented for the proposed application of the test, the degree to which it claims to assess a construct (Cordeiro et al., 2020). Convergent validity refers to how closely the new scale is related to other measurements and variables of the same construct (Krabbe, 2017). Divergent validity investigates whether structures that are supposed to be unrelated are actually unrelated. For validity testing, we used Spearman's correlation coefficient *r* to measure a relationship between the Portuguese Brazilian versions of the short-version-PDS and DMS-5 scales. The correlation intensity was rated as negligible (0.30), low (0.30 to 0.50), moderate (0.51 to 0.70), high (0.71 to 0.90), and very high (>0.90) (Mukaka, 2012).

Diagnostic utility was evaluated using the area under the receiver-operating characteristic (ROC). To measure the diagnostic accuracy, the following indices were used: area under the curve (AUC) with standard error (SE) and its binomial exact 95% confidence interval; Youden's J index; sensitivity (Sn); specificity (Sp). Spearman's correlation coefficient and ROC were done using GraphPad Prism version 6.0e for Mac OS X, GraphPad Software, La Jolla California USA, www.graphpad.com.

## Results

### Translation and Cross-Cultural Adaptation Process of the Short-Version-PDS

The final version of the Brazilian Short-Version-PDS is attached as Supplementary Material 1.

In the pre-test, the experts indicated the synthesis for the three items evaluated as the best representation with CVC coefficients > 0.70 (Table 1). Although translation 2 of item 1 reached a coefficient above the cutoff point, the synthesis in this item and in the others reached the highest score. If the coefficients are below the cutoff point, this item must be reformulated and sent to the judges again. As our goal was to find the best translation, we found the one with the highest agreement rate. Therefore, the translation that best suits the characteristics of PTSD, consisted of the translation synthesis. The calculation of the total content validity (CVCt) also demonstrated that the synthesis for all items is more suitable for test applications. This is because the experts mostly agreed that this item is the one that best represents PTSD. Item 1 presented CVCt = 0.92; item 2 had a CVCt = 0.87 and item 3 had a CVCt = 0.95.

**Table 1:** Brazilian translation and synthesis of the three Short-Version-PDS items.

**Item 1**		**Mean**	**CVC _**total**_**
Translation 1	Mesmo não querendo você se pega pensando/ imaginando no seu trauma.	0.776667	0.776346667
Translation 2	Embora eu não queira, meus pensamentos e imagens sobre eventos traumáticos vêm à minha mente e me aborrecem.	0.853333	0.853013333
Translation 3	Pensamentos e lembranças indesejáveis do evento traumatizante.	0.686667	0.686346667
**Synthesis**	**Mesmo não querendo, tenho pensamentos e lembranças indesejáveis do evento traumático e isso me aborrece**	**0.923333**	**0.923013333**
**Item 2**		**Média**	**CVC**_**total**_
Translation 1	Você tem pesadelo com o seu trauma.	0.796667	0.796346667
Translation 2	Eu tenho um sonho desagradável ou pesadelo sobre um evento traumático.	0.653333	0.653013333
Translation 3	Sonhos ruins e perturbadores com o evento traumatizante.	0.69	0.68968
**Synthesis**	**Tenho sonhos ruins e perturbadores com o evento traumático**.	**0.876667**	**0.876346667**
**Item 3**		**Média**	**CVC**_**total**_
Translation 1	Quando te fazem lembrar o seu trauma você tem reações física (por exemplo; suor, coração acelerado, falta de ar).	0.626667	0.626346667
Translation 2	Quando me lembrei de um evento traumático, experimentei uma resposta fisiológica (por exemplo, suor, o coração estava batendo).	0.786667	0.786346667
Translation 3	Ter reações físicas intensas quando me lembro do acontecimento traumático ou de algo relacionado (por exemplo, coração batendo rápido, suor excessivo etc.)	0.766667	0.766346667
**Synthesis**	**Quando me lembro do acontecimento traumático ou de algo relacionado, tenho reações f**í**sicas intensas, como por exemplo, coração batendo rápido, suor excessivo, falta de ar etc**.	**0.96**	**0.95968**

For the analysis of the final version, reliability was performed using Cronbach's alpha (0.78), which proved to be statistically significant.

### Correlation Between the Translated Short-Version-PDS and the DMS-5 Scale

There was a strong positive correlation between the two Portuguese Brazilian versions of the short-version-PDS and DMS-5 scales as indicated by a Spearman's rho of 0.64 (95%CI: 0.44 to 0.78).

### Prediction Value of the Translated Short-Version-PDS for the Severity of PTSD

**Figure 1:**
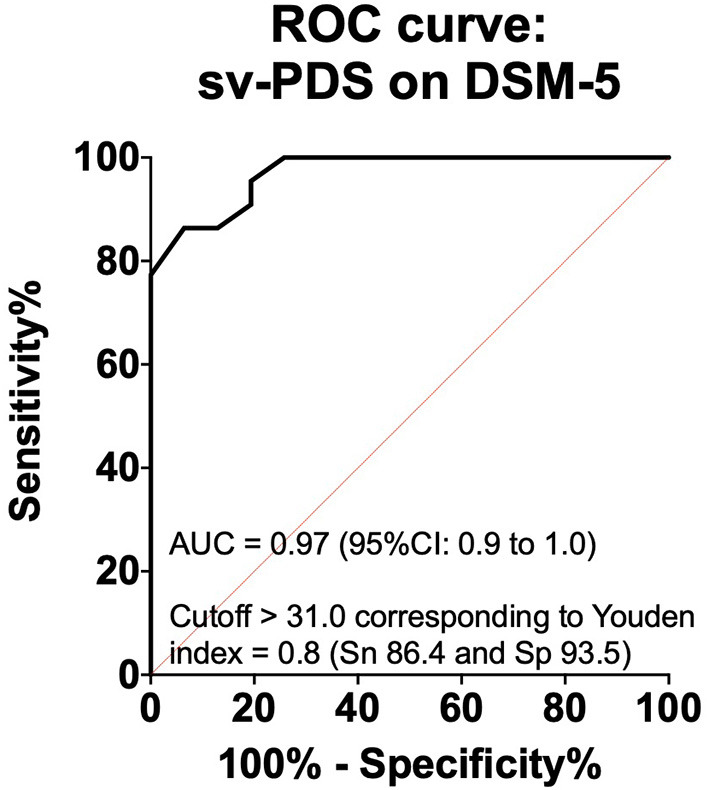
Receiver operating characteristic (ROC) curve for sv-PDS (short version of the Posttraumatic Diagnostic Scale) for the diagnosis of PTSD.

The calculated area under a ROC curve was 0.97 ± 0.02 with significance value of *P* < 0.0001 (Figure 1). The short-version-PDS is good at distinguishing between patients with aggravated (mild, moderate, and severe) and those with slight disease diagnosed with DSM-5 score, with a cutoff value > 31 and a sensitivity prediction of 86.4% and specificity 93.5%.

## Discussion

The results of this study showed the psychometric adequacy of Brazilian Portuguese translation of a Japanese short-version-PDS, suggesting that the instrument is adequate for use in Brazilian contexts. This Brazilian translation and cross-cultural adaptation of the Japanese short-version-PDS was found to have good reliability, validity, and diagnostic utility.

The goal of the content analysis was to analyze the cross-cultural adaptation and content validity of the Japanese short-version-PDS 3-item scale as a tool for assessing PTSD in Brazilian population. The Japanese short-version-PDS was compared to the Japanese PTSD checklist for DSM-IV (PCL) (which are the same as those in the DSM-5) (Asukai et al., 2003; Itoh et al., 2017a). Similarly, in this study, we compared PTSD diagnosis using the Brazilian version of the latest short-version-PDS to PTSD diagnosis using the Brazilian version of the PCL-5, which has demonstrated high reliability and diagnostic utility for PTSD (Pereira-Lima et al., 2019).

The translated version reached an adequate degree of agreement by the expert panel when assessed its equivalence in terms of several criteria (i.e., semantic, linguistic, cultural, conceptual, clarity, and precision) through a quantitative measurement (CVC calculation). The final structure of the content analysis consistency with the original scale allowed subjects with PTSD to be evaluated in the Portuguese-speaking context.

The volunteers in this study had a direct exposure to a traumatic incident as described by DSM-5 Criterion A for PTSD, which included medical conditions such as cardiovascular (Remch et al., 2018), oncology (Cordova et al., 2017), and obstetrics and gynecology (Canfield and Silver, 2020), as well as work-related events (Giorgi et al., 2020). When tested on volunteers with at least one potentially traumatic experience, the Brazilian short-version-PDS had a strong positive correlation with the validated Brazilian DMS-5 scale in diagnostic abilities for PTSD between, as indicated by Spearman's rho and ROC curve, and defined as strong correlation > 0.60 in behavioral sciences (Cohen, 1988). This indicates that the new Brazilian short-version-PDS has convergent validity, that is, that the two instruments measure the same theoretical construct, that is, they measure Post Traumatic Stress Disorder.

Public health crises such as COVID-19 pandemic have provided significant challenges for mental health care systems. In such crises, reconsidering ways of working in a rapid time scale are of major interest (Smith et al., 2020). During such public health crises, developing and applying valid short screening tools are necessary to identify high-risk groups for posttraumatic stress disorder (PTSD) (Itoh et al., 2017a).

## Limitations of the Study

There are several limitations that should be taken into consideration. Although the Brazilian short-version-PDS demonstrated content validity, it is not without limitations, one of which is that it needs to be applied to a larger sample for a factorial validity. Due to the sample size, the results of this study could be deemed as “preliminary results.” Since the participants were drawn from a single hospital, they could not represent all Brazilians. Furthermore, due to practical constraints, we did not assess the test–retest reliability. Finally, in order to demonstrate more proof of validity, we must conduct additional research to determine if the short-version-PDS and DSM-5 PTSD scales are linked to a specific factor (e.g., emotional exhaustion, tension, anxiety) and if there is reciprocity in the existing relationships.

## Conclusion

This study indicates that the Brazilian short-version-PDS had good psychometric properties among Brazilian adults with and without PTSD. This scale showed internal consistency, validity and diagnostic utility. Follow up studies will allow to screen patients while minimize contact (self-rating), as is common with internet surveys. Transferability and generalizability of the cut-off scores should be further analyzed.

## Data Availability Statement

The raw data supporting the conclusions of this article will be made available by the authors, without undue reservation.

## Ethics Statement

The studies involving human participants were reviewed and approved by Medical Ethics Committee of Anhembi Morumbi University. The patients/participants provided their written informed consent to participate in this study.

## Author Contributions

CdF, LC, and OB: study conception and design. CdF, and NO: performed the study. CdF, VT, TP, LC, and OB: assays and data analysis. CdF, NO, VT, TP, LC, and OB: interpretation of the data. OB and LC: writing of the manuscript. CdF, NO, VT, and TP: critical revision of the manuscript regarding the important intellectual content. All authors contributed to the article and approved the submitted version.

## Conflict of Interest

The authors declare that the research was conducted in the absence of any commercial or financial relationships that could be construed as a potential conflict of interest.
