# Supplementary material for: Cross-Cultural Adaptation, Reliability, and Validity of a Brazilian of Short Version of the Posttraumatic Diagnostic Scale
Source: Front Psychol. 2021 Apr 23;12:614554. doi: 10.3389/fpsyg.2021.614554 (PMC8102692; doi:10.3389/fpsyg.2021.614554)
Supplement: Supplementary file 1 [file Data_Sheet_1.docx]

**Escala de Diagnóstico Pós-Traumático**

*Instrumento simplificado para triagem*

**Nome:** __________________________________________________________

**Idade:**  __________ **Sexo:** ( ) Feminino ( ) Masculino

**Data da Avaliação:** ______/ ______/ ________

**Marque uma ou mais situações estressantes que você vivenciou até o dia de hoje:**

Caso não tenha vivenciado nenhum dos itens a seguir, não é necessário assinalar!

1. □ Acidente grave (Incêndio, explosão, acidente de carro, acidente de avião etc.)
2. □ Desastre natural (Deslizamento de terra, inundação, terremoto, tornado, furacão etc.)
3. □ Agressão física (Espancamento, assalto, tiros, ameaça com arma branca etc.)
4. □ Violência sexual (Estupro, tentativa de estupro, ameaça com o uso de uma arma para um ato sexual etc.)
5. □ Doença que ameace a vida (Ataque cardíaco, diagnóstico de câncer potencialmente fatal etc.)
6. □ Abuso infantil (Espancamento, encarceramento, abandono, ameaça de morte, violência com castigo corporal, situação com perigo de morte, testemunha de violência contra pessoas próximas etc.)
7. □ Experiência de luta corporal, exposição a uma área de guerra ou conflito
8. □ Tortura, cativeiro
9. □ Morte súbita de alguém próximo, avistamento de um corpo ferido, notícia de morte de uma pessoa próxima
10. □ Outro ________________________________________________________________

**Nos últimos 30 dias, em quais das situações você teve ou está tendo mais dificuldade?**

Escreva o número _____________

(Caso tenha assinalado apenas um item, considere ele para responder as questões a seguir)

**Quantos anos você tinha quando o evento aconteceu?** __________anos _________meses

**Quantos anos você tinha quando o evento terminou?** (Caso o evento tenha sido duradouro)

________anos _________meses

**Considerando os últimos 30 dias e a situação que você teve ou está tendo mais dificuldade, circule o número (de 0 a 3) para a resposta que seja mais adequada:**

| **Mesmo não querendo, tenho pensamentos e lembranças indesejáveis do evento traumático e isso me aborrece** | | | |
| --- | --- | --- | --- |
| **0** | **1** | **2** | **3** |
| Nenhuma vez/ Apenas uma vez | Menos de uma vez por semana/ Às vezes | 2 a 4 vezes por semana/ Quase metade do tempo | 5 ou mais vezes por semana/ Quase sempre |

| **Tenho sonhos ruins e perturbadores com o evento traumático** | | | |
| --- | --- | --- | --- |
| **0** | **1** | **2** | **3** |
| Nenhuma vez/ Apenas uma vez | Menos de uma vez por semana/ Às vezes | 2 a 4 vezes por semana/ Quase metade do tempo | 5 ou mais vezes por semana/ Quase sempre |

| **Quando me lembro do acontecimento traumático ou de algo relacionado, tenho reações físicas intensas, como por exemplo, coração batendo rápido, suor excessivo, falta de ar etc.** | | | |
| --- | --- | --- | --- |
| **0** | **1** | **2** | **3** |
| Nenhuma vez/ Apenas uma vez | Menos de uma vez por semana/ Às vezes | 2 a 4 vezes por semana/ Quase metade do tempo | 5 ou mais vezes por semana/ Quase sempre |
